# Accurate Identification and Quantification of Chinese Yam Powder Adulteration Using Laser-Induced Breakdown Spectroscopy

**DOI:** 10.3390/foods11091216

**Published:** 2022-04-22

**Authors:** Zhifang Zhao, Qianqian Wang, Xiangjun Xu, Feng Chen, Geer Teng, Kai Wei, Guoyan Chen, Yu Cai, Lianbo Guo

**Affiliations:** 1 School of Optics and Photonics, Beijing Institute of Technology, Beijing 100081, China; 3120215335@bit.edu.cn (Z.Z.); 3120205322@bit.edu.cn (X.X.); 3120185341@bit.edu.cn (G.T.); 3120170346@bit.edu.cn (K.W.); 3120190515@bit.edu.cn (G.C.); 2 Key Laboratory of Photonic Information Technology, Ministry of Industry and Information Technology, Beijing Institute of Technology, Beijing 100081, China; 3 Yangtze Delta Region Academy of Beijing Institute of Technology, Jiaxing 314033, China; 4 Wuhan National Laboratory for Optoelectronics, Huazhong University of Science and Technology, Wuhan 430074, China; feng_chen@hust.edu.cn; 5 College of Pharmacy, Hubei University of Chinese Medicine, Wuhan 430065, China; yucai2018@hbtcm.edu.cn

**Keywords:** Chinese yam powder adulteration, identification and quantification, laser-induced breakdown spectroscopy, random forest-support vector machine, Gaussian process regression

## Abstract

As a popular food, Chinese yam (CY) powder is widely used for healthy and commercial purposes. Detecting adulteration of CY powder has become essential. In this work, chemometric methods combined with laser-induced breakdown spectroscopy (LIBS) were developed for identification and quantification of CY powder adulteration. Pure powders (CY, rhizome of winged yam (RY) and cassava (CS)) and adulterated powders (CY adulterated with CS) were pressed into pellets to obtain LIBS spectra for identification and quantification experiments, respectively. After variable number optimization by principal component analysis and random forest (RF), the best model random forest-support vector machine (RF-SVM) decreased 48.57% of the input variables and improved the accuracy to 100% in identification. Following the better feature extraction method RF, the Gaussian process regression (GPR) method performed the best in the prediction of the adulteration rate, with a correlation coefficient of prediction (R_p_^2^) of 0.9570 and a root-mean-square error of prediction (RMSEP) of 7.6243%. Besides, the variable importance of metal elements analyzed by RF revealed that Na and K were significant due to the high metabolic activity and maximum metal content of CY powder, respectively. These results demonstrated that chemometric methods combined with LIBS can identify and quantify CY powder adulteration accurately.

## 1. Introduction

Chinese yam (*Dioscorea oppositifolia* L., CY), the rhizome of dioscorea opposite thumb, is a food crop in East Asia and West Africa [1], containing proteins, starches, vitamins, and other nutrients essential to the human body. Besides, CY is a widely-used ingredient in traditional Chinese medicine because it has many roles such as anti-tumor, anti-oxidation, and anti-inflammation from its polysaccharides, flavonoids, polyphenols, steroidal sapogenins, etc. [2]. With the popularity in people, the global production of CY was increased dramatically from 17 million tons in 1988 to 73 million tons in 2018, and it ranks as the fourth most important tuber crop in economic terms [1,3].

CY powder, as a kind of health-care food with more convenient intake and better absorption by breaking cell walls, has been demanded increasingly by consumers recently [4]. Faced with strong market demands, some businesses sell adulterated CY powder to gain large profits. The common adulteration is CY powder mixed with powders of similar species and looking, including rhizome of winged yam (*Dioscorea alata* L., RY) and cassava (*Manihot esculenta* Crantz, CS) [5,6], whose prices are 1/2 to 1/6 of CY powder, respectively. The adulteration phenomena disrupted market security and authority, because these adulterants changed the nutrients and officinal ingredients of CY powder, which resulted in financial loss, inefficient function, allergic reactions, and even poisoning accidents [7]. Therefore, the adulteration detection of CY powder is an important issue for both consumers and producers. Common adulteration detection methods for CY powder include microscopic observation [8], chromatographic techniques [9], mass spectrometry [10], and so on. These methods have disadvantages, such as complex preprocessing, long detection time, high technical requirements and complicated equipment maintenance. Recently, some researches have reported that near-infrared spectroscopy could be applied to the adulteration detection of CY powder [7,11,12], but it still has the disadvantages of expensive cost, high requirement for background light, and poor anti-interference ability.

Laser-induced breakdown spectroscopy (LIBS), a reliable technique for emission spectroscopy analysis, has been applied in geological prospecting [13], industrial monitoring [14,15] and tissue identification [16]. With the merits of simple sample pretreatment, in situ detection and real-time analysis [17], LIBS is gradually becoming popular in the detection of adulteration. Dimitrios Stefas et al. investigated the effects of artificial feeding of bees on the honey using LIBS combined with LDA and RF, and evaluated the importance of metal elements for classification, with accuracies more than 90%. More precise quantification of mixed categories is needed [18]. Banu Sezer et al. identified the beef, chicken, and pork in fermented sausage and salami products using protein-based LIBS, and the limit of detection values by partial least square analysis model were 3.68%, 3.83%, 3.80% and 3.47%, respectively [19]. The measured content rates of validation are within calibration, and the rates in unknown conditions require further verification. Weihua Huang et al. used CNN to classify the adulterated milk powder mixed with four different types of exogenous proteins from the range of 5–20%, and its average accuracy was 97.8% [20]. The adulteration range can be further extended for verification. While the references above have made significance to adulteration detection by LIBS, there are few studies roundly conducted on qualitative and quantitative adulteration using LIBS, let alone CY powder adulteration. Meanwhile, the identification accuracy and prediction precision of adulteration studies need to be further improved.

In this work, we distinguished CY powder from its adulteration using LIBS combined with a random forest-support vector machine (RF-SVM) model and quantified the ratio of adulterants in CY with a RF-Gaussian process regression (GPR) model by LIBS. CY, RY and CS slices were ground into powder for tableting in LIBS spectral collection. Based on the results of RF method, the spectral lines were ranked and the input number was optimized to improve the identification accuracy and prediction precision. Some common indexes were used to evaluate the performance for discrimination and quantitative models.

## 2. Materials and Methods

### 2.1. Experiment Setup

**Figure 1:**
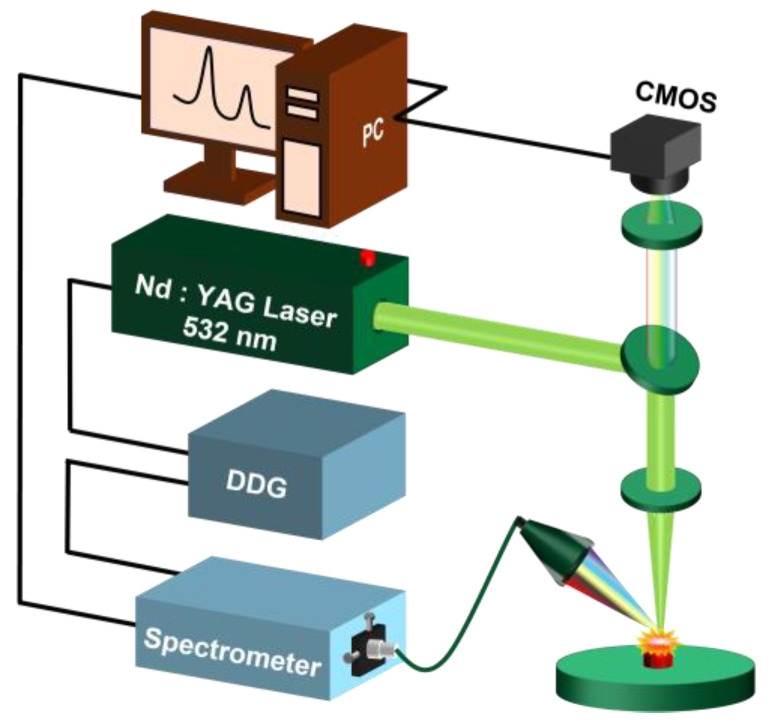
Schematic diagram of the LIBS setup (PC: personal computer; DDG: digital delay generator).

The schematic diagram of the LIBS system is shown in Figure 1. The hardware device of the LIBS system mainly comprised a Q-switched Nd: YAG laser (wavelength: 532 nm; pulse width: 8 ns; flattened Gaussian beam; Beamtech Optronics Co., Ltd., Beijing, China, Nimma-400), a 45° plate beam splitter (350–1100 nm R: T = 50:50), a quartz lens (f = 150 mm), a six-channel fiber-optic spectrograph (Avantes B.V., Apeldoom, Netherlands, AvaSpec-ULS4096CL-EVO, spectral ranges: 196–874 nm, minimum gate width: 9 μs), a digital delay generator (DDG, Stanford Research Systems, Sunnyvale, CA, USA, DG645), a CMOS (Thorlabs, Newtown, New Jersey, USA, DCC1545M, resolution: 1280 × 1024 pixels), an XYZ motion platform (Beijing Jiangyun Juli Technology, Beijing, China, DZY110TA-3Z), a collector and a computer. More device details can be found in a previous study [21]. The laser energy, gate width, and delay time were set to be 35 mJ, 9 µs and 1 µs, respectively. Each spectrum was the average of 5 pluses.

### 2.2. Sample Pretreatment

Dry slices of Chinese yam (CY), cassava (CS), and rhizome of winged yam (RY) were authenticated and provided by Hubei University of Chinese Medicine. CY is from Wen County, Henan Province in China, which belongs to one of the most popular varieties tiegun yam, famous for its nutrients and active ingredients [22]. CS and RY are from Xiangtan City, Hunan Province and Nanning City, Guangxi Province in China, respectively, and they are common varieties in the market. These slices were placed in a 60 °C drying baker around 6 h until the quality had no change, and they were ground into powder to pass through a 100-mesh sieve. According to the market research, the price of CS is lower than that of RY, so incorporating CS into CY can better meet the interests of illegal traders. Pure powders (CY, CS and RY) and CY powder adulterated by CS powder in the range of 0–100% at a 5% gradient were used for identification and quantitation tests, respectively. The details of samples are shown in Table 1. Various powders (1 g) assisted by boric acid powder (Sinopharm Chemical Reagent Co., Ltd., Shanghai, China; 9 g) were pressed into pellets of 40 mm diameter by a pressure of 30 tons. For each category, 3 pellets were made to do the repeated experiments. In total, 72 pellets were prepared for experiment.

### 2.3. Algorithm Description

The feature extraction methods used in this paper include principal component analysis (PCA) and random forest (RF). PCA eliminates possible multicollinearity between variables based on variance in projection [23], obtaining the contribution rate of each component depending on the eigenvalue ratio. RF gives estimation of variable importance based on the Gini coefficient or out-of-bag error in the classification [24]. They can be used for feature extraction according to the contribution rate of components or importance of variables (collectively called significance of features). The significance of the ith (i=1⋯m,⋯n) feature is *I_i_*, and the accumulative significance $AI_{m}$ of the 1th−mth features is calculated according to Equation (1).

(1) $$AI_{m}=\sum_{i=1}^{m}I_{i}/\sum_{i=1}^{n}I_{i},(i=1,\cdots m,\cdots n)$$

K-nearestneighbor (kNN), decision tree (DT), naïve bayes (NB), and support vector machine (SVM) classifiers are widely used for matter identification [17,25,26,27]. The partial least-square regression (PLSR), ensemble machine learning (EML), linear regression (LR) and Gaussian process regression (GPR) are widely used for chemical component prediction [8,28,29,30].

In this work, we applied these algorithms to detect CY powder adulteration firstly. The spectral data collected by the spectrometer were saved into csv files by LIBSsystem software written by laboratory personnel [31]. Python 3.7.0 platform was used to read and process data in csv files.

**Figure 2:**
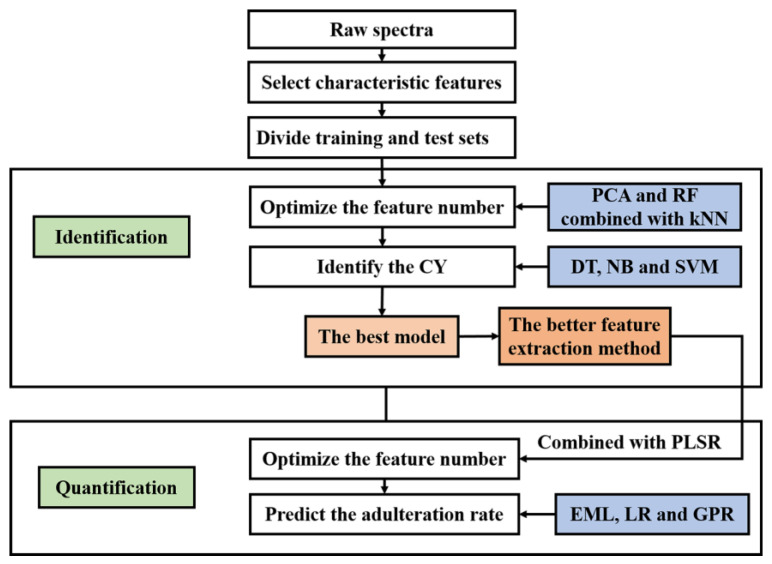
The details of data processing (CY: Chinese yam; PCA: principal component analysis; RF: random forest; kNN: k-nearestneighbor; DT: decision tree; NB: naïve bayes; SVM: support vector machine; PLSR: partial least-square regression; EML: ensemble machine learning; LR: linear regression; GPR: Gaussian process regression).

The details of data processing are shown in Figure 2. The steps of spectral data processing are performed as follows.

1. Find the characteristic element peaks of CY LIBS spectra.
2. Divide the training set and test set of LIBS spectra for classification samples randomly by the proportion 2:1.
3. Use PCA and RF combined with kNN to optimize the number of inputs for identification, respectively.
4. With the optimized features, train the DT, NB and SVM classifiers, and identify CY by different models, respectively.
5. With the better feature extraction method, optimize the number of features combined with PLSR for quantification.
6. Train the LR, EML and GPR models, and quantify CY adulteration by different models, respectively.
7. For evaluation indexes, the recognition accuracy, the root-mean-square error (RMSE) and the correlation coefficient were used for identification and quantitation, respectively.

## 3. Results and Discussion

### 3.1. Spectral Analysis

**Figure 3:**
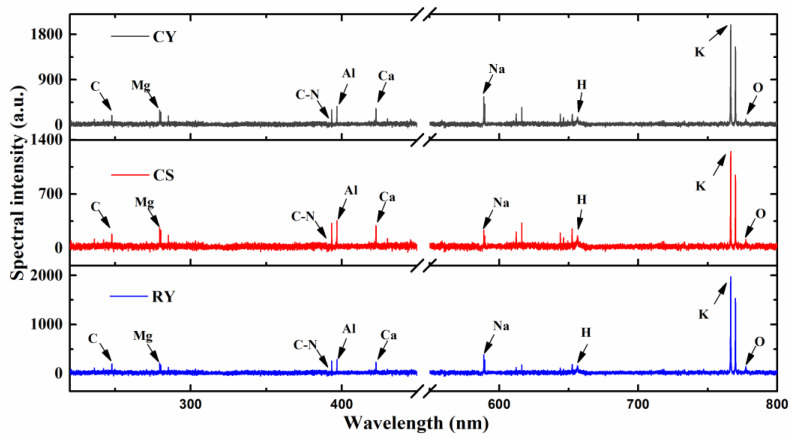
The LIBS spectra of CY, CS and RY samples.

With the described setup and optimized parameters, 720 and 1260 spectra were obtained from qualitative and quantitative samples, respectively. The LIBS spectra ranging from 220 nm to 800 nm are shown in Figure 3.

Elements including C, H, O, K, Ca, Na, Mg, Al and molecular bands C-N could be observed. The spectra of CY, CS and RY have the same trend: their peaks were located in the same position but have different intensities, which implied they have the same element variety but different contents. Among these peaks in CY, the peak of the K element line has the highest intensity, because CY has the strongest ability to enrich element K from soil [32]. The peak intensity of Na element line ranked the second in CY, but this ranking did not match CS. There are many similar analyses for different elements, and they provide the basis of CY powder adulteration detection.

Due to the large spectral dimension (24564 bands), it is necessary to reduce redundant information. The characteristic lines were selected initially for facilitate identification and quantification, and the details of 35 selected spectral lines for CY adulteration analysis are shown in Table 2.

### 3.2. Identification of CY Adulteration

**Figure 4:**
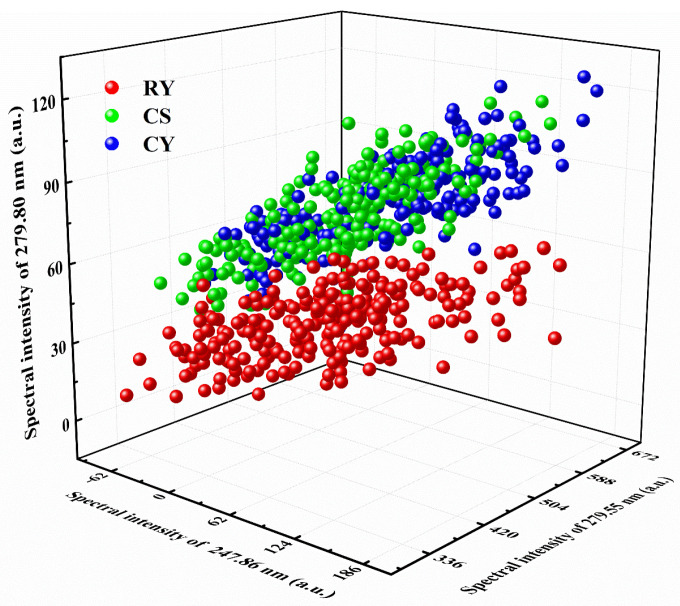
The visualization of raw features for CY, CS and RY.

The 3D visualization of the selected features for CY, CS and RY powders was shown in Figure 4. The CY data were almost mixed with the CS data completely, and there were some overlapping areas between the CY, CS and RY data, which suggested that it was difficult to identify CY using simple classification methods. It is necessary to extract features further and find an appropriate classifier for accurate classification.

**Figure 5:**
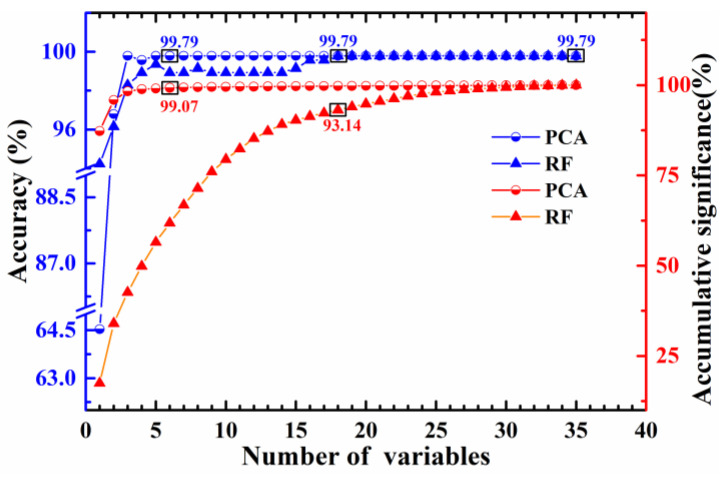
The optimization process of feature number for PCA and RF.

After dividing the training and test sets, the optimal feature set of the training set needed to be extracted. To reduce the redundant information, PCA and RF were applied to acquire the optimal feature subset for feature extraction. For PCA, the scores of principal components (PCs) were extracted as feature variables. For RF, the intensities of spectral lines were selected as feature variables. After the contribution rate of each PC was assessed by PCA, the PCs were sorted by the contribution rate. Similarly, the importance of each spectral line was evaluated by RF, these spectral lines were sorted in order. Combined with the kNN classifier (The k value was set to 1, and the distance was calculated by Euclidean distance.), different numbers of features were used as input variables to identify the CY. Hence, a series of accuracies could be obtained, and the feature number was determined by the highest accuracy. The optimization process of feature extraction is shown in Figure 5. As the number of features increasing, the float of the accumulative significance became smaller and smaller, and the curves became even. The advantage of PCA is to remove redundant information, so the accumulative significance was relatively stable and the accuracy was high when the number of components was small. RF has the merit of evaluating the importance of variables without data compression, so the high accuracy and flat trend appeared relatively slowly. The accuracy of the kNN classifier combined with RF was 99.79% by 35 raw features, that was, the accuracy was 99.79% by all features. After reducing the dimension by PCA, with the first 5 PCs, the highest accuracy of kNN classifier was 99.79%, and the accumulative significance of the first 5 PCs was 99.07%. For the RF method, with 18 characteristic lines, the accuracy reached the maximum 99.79%, and the accumulative significance was 93.14%. The highest accuracy of kNN-PCA was the same as that of kNN-RF, and the accuracy was essential to be improved further.

**Figure 6:**
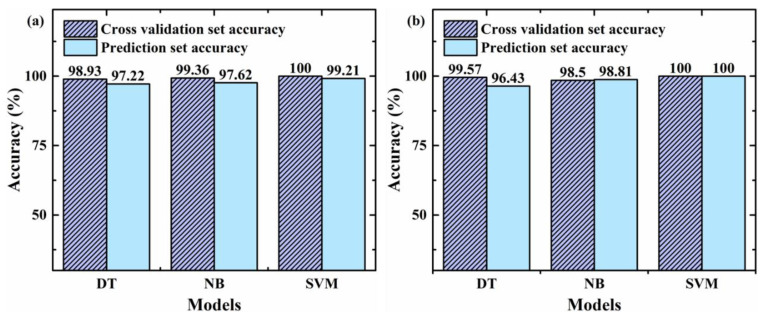
The CY powder identification results of different models, (**a**) PCA feature extraction, and (**b**) RF feature extraction (DT: decision tree; NB: naïve bayes; SVM: support vector machine).

To avoid the result limitation of a single classifier kNN, the optimal feature subsets from PCA and RF were applied to DT (The algorithm is CART), NB and SVM (The kernel was optimized to the linear kernel.) to recognize CY, respectively. The classification results of different models are shown in Figure 6. From PCA to RF for feature extraction, the prediction set accuracies of DT, NB and SVM ranged from 97.22%, 97.62% and 99.21% to 96.43%, 98.81% and 100%, respectively. Among them, the RF-SVM model performed best, with the highest accuracy of 100%. This result indicated that RF was more suitable than PCA for the detection of CY adulteration. Compared with PCA, RF retains the original value of each feature according to the variable importance, and it is possible to retain the nonlinear relationship between features to obtain the best result. For the three classifiers, DT had the worst classification result, because DT summarizes a set of classification rules from the training set by selecting samples randomly, probably leading to the repeated selection of some samples, resulting in a local optimal solution. NB performed slightly worse than the SVM model. NB assumes that the input conditions are mutually independent, ignoring the correlation between variables. While the aim of SVM is to maximize the marginal distance between two categories, it makes the optimal solution possible.

In the best model RF-SVM, the importance sequence of 18 variables is listed in Table 3. Except Mg 279.80 is the ionic line, the other 17 variables are all atomic spectral lines. The roles of metal elements are more important than non-metal elements, and the importance sequence of metal elements was Na 589.00 nm > K 769.90 nm > Mg 518.36 nm > Ca 616.22 nm > Al 396.15 nm. Element Na is the most important element in the classification, because it is widely involved in plant metabolism, such as osmotic regulation, water metabolism and nutrient transport [33]. The importance of Na in CY has been proven in relevant studies, which is one of the reasons for the high metabolic activity of CY [34]. Furthermore, element K maintains resistance to cold, drought and disease, participating in stomatal regulation, synthesis of essential compounds and sucrose transportation. It can be inferred that element K is related to the high contents of resistant starches, polysaccharides and steroidal saponins in CY [1]. Elements Mg and Ca are related to chlorophyll and cell walls, which involves the differences of starches and fiber contents, respectively [35]. Therefore, the spectra of metal elements are vital to the identification of CY powder adulteration.

Overall, with the best model RF-SVM, the input variables were decreased by 48.57% and the recognition accuracy was improved from 99.75% to 100%. The results indicated that it is feasible to identify CY powder adulteration accurately using LIBS combined with RF-SVM model. Meanwhile, the accuracy showed the advantage of RF in estimation of variable importance for CY powder adulteration detection. Therefore, RF could be applied to predict the adulteration rate of CY powder further.

### 3.3. Quantification of the Adulterants in CY Powder

Based on the accurate CY identification, it is necessary to quantify the CY powder adulteration rate further. Meanwhile, RF will continue to be used in the quantitative adulteration of CY powder.

**Figure 7:**
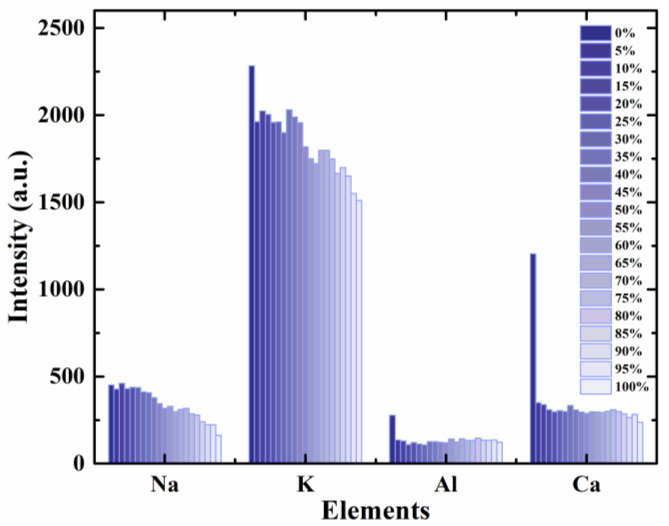
The spectra intensities of four elements in adulterants with different gradients.

For 35 raw features from the calibration set, RF combined with PLSR was used to optimize the number of feature subsets in the regression. Similar to the optimization process in CY powder identification, when the number of the features was 13, the R_p_^2^ was the largest and the RMSEP was the smallest. These 13 features listed in Table 4 are all atomic lines. They included four elements Na, K, Al and Ca, and the sorting result of atomic lines for these 4 metal elements was Na 589.00 nm > K 769.90 nm > Al 396.15 nm > Ca 616.22 nm. Compared with adulteration identification, there is no spectrum of element Mg for quantitative analysis, and the importance of Al and Ca switched order in quantification. This result suggested that Mg might played an important role in RY to a certain extent. To explore the variation trend of elements in CY quantitative adulteration further, the average spectral intensities of these four elements (Na, K, Al and Ca) lines in 0–100% adulterated samples were counted and is shown in Figure 7. For each element, the spectra intensities of 0% (pure CY) and 100% (pure CS) adulterated samples corresponded to the highest and the lowest, respectively. After doping CS, the spectral intensity in different gradients for the four elements did not show an obvious linear relationship. The phenomenon might mainly result from unavoidable matrix effect [36], that is, except for the test substance CS, the overall composition changed with the amount of CS added. Among these elements, the spectral intensities of the K and Ca lines had a larger fluctuation range than those of the Na and Al lines, which are related to the differences in osmotic balance and cell hardness between CY and CS, respectively [35].

After feature extraction by RF, a regression method was needed to predict the proportion of adulteration accurately. LR is the basic method of quantitative relationship among statistical variables [37]. EML combines multiple weakly supervised models to obtain a more comprehensive supervised model (The ensemble method in this article is optimized to bagging regression trees, including 30 trees.) [38]. The essence of GPR is probabilistic reasoning (The kernel is optimized to Matern kernel.). EML and GPR are rarely used in LIBS, and they have gradually received attention recently in LIBS [25,39]. With 13 features, EML, LR and GPR were applied to predict the adulteration rate. The results of different models are listed in Table 5.

**Figure 8:**
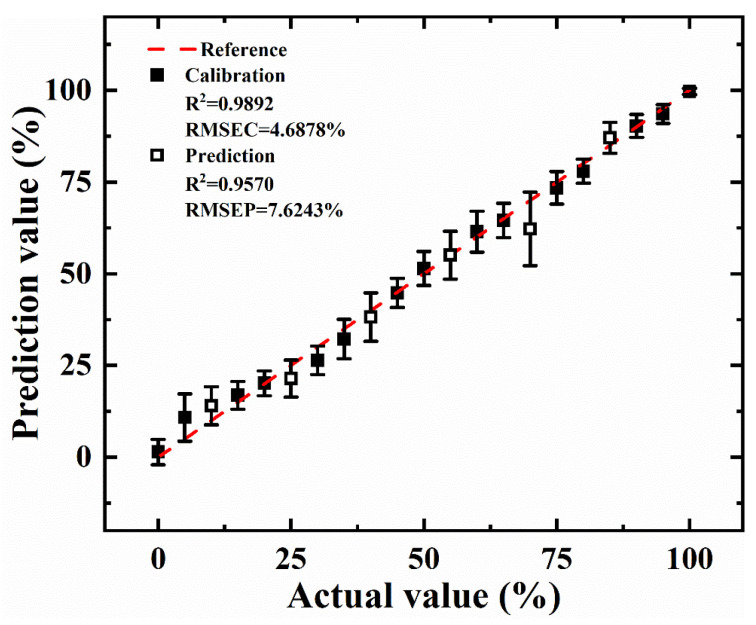
The prediction result of GPR model.

Among them, the GPR model performed the best with R_c_^2^ of 0.9892, RMSEC of 4.6878%, R_p_^2^ of 0.9570 and RMSEP of 7.6243%. Compared with EML and LR, the superiority of GPR is that it can quantify the uncertainty of prediction in a principled way, so the prediction result of GPR was the best. More prediction details of GPR are shown in Figure 8. The predicted values of the calibration set and prediction set were highly linear with the reference values, and the dispersion of each predicted gradient was relatively small.

The results demonstrated that the RF-GPR model could predict the extent of adulterants in CY across a wide range of gradients accurately. It is feasible to predict the percentage of adulteration in CY powder using LIBS with the RF-GPR model.

## 4. Conclusions

This work aimed to distinguish CY powder from confused substances (CS and RY) and quantify the adulterant (CS) in CY powder accurately using LIBS combined with the RF-SVM model and RF-GPR model, respectively. For CY powder discrimination, PCA and RF combined with kNN were used to extract the feature variables and optimize the feature number, respectively. With the optimized features, the RF-SVM model had the best recognition accuracy of 100%, and the input variables were decreased by 48.5%. For adulterant quantification, RF-GPR model performed the best with R_c_^2^ of 0.9892, RMSEC of 4.6878%, R_p_^2^ of 0.9570 and RMSEP of 7.6243%. Moreover, the analysis of variable importance for metal elements revealed that Na and K played important roles in the identification and quantification of CY powder adulteration, which were related to the high metabolic activity and maximum metal content of CY, respectively. The results verified that the RF-SVM and RF-GPR models are effective methods for LIBS analysis in CY powder adulteration. Therefore, chemometric methods combined with LIBS can be a practical tool for accurate detection of CY powder adulteration.

## Figures and Tables

**Table 1:** The details of sample preparation.

Experiment Type	Sample Type	Preparation Method	Quantity
Qualitative	Training set and test set	Pure CY	3
		Pure CS	3
		Pure RY	3
Quantitative	Calibration set	0%, 5%, 15%, 20%, 30%, 35%, 45%, 50%, 60%, 65%, 75%, 80%, 90%, 95%, 100%	45
	Validation set	10%, 25%, 40%, 55%, 70%, 85%	18

**Table 2:** Characteristic lines used for CY powder adulteration analysis.

Element	Wavelength (nm)	Element	Wavelength (nm)
C-N	386.19, 387.14, 388.34	K	404.41, 766.49, 769.90
C	247.86	Na	285.28, 589.00, 589.60, 819.48
H	656.29	Mg	279.55, 279.80, 280.27, 517.27, 518.36
O	777.19, 777.42, 777.54	Al	396.15
Ca	422.67, 442.54, 443.50, 443.57, 445.48, 445.59, 445.66, 558.88, 610.27, 612.22, 616.22, 643.91, 646.26, 649.38		

**Table 3:** The significance sequence of 18 features in the RF-SVM model.

Sequence Number	Element Line (nm)	Sequence Number	Element Line (nm)
1	Na 589.00 *	10	Ca 443.50
2	Na 589.60	11	Ca 643.91
3	K 769.90 *	12	K 404.41
4	Mg 518.36 *	13	Ca 646.26
5	Ca 616.22 *	14	Ca 445.48
6	Al 396.15 *	15	O 777.54
7	Mg 279.80	16	O 777.19
8	Ca 612.22	17	Na 285.28
9	Ca 610.27	18	O 777.42

Note: * means the line appearing first in the same metal element.

**Table 4:** The significance sequence of the 13 optimized features.

Sequence Number	Element Line (nm)	Sequence Number	Element Line (nm)
1	Na 589.60	8	Ca 643.91
2	Na 589.00 #	9	K 766.50
3	Na 819.48	10	Ca 558.88
4	K 404.41	11	Ca 610.27
5	K 769.90 #	12	Ca 612.22
6	Al 396.15 #	13	Ca 422.67
7	Ca 616.22 #		

Note: # means the metal element line with * in the Table 3.

**Table 5:** The results of different models for adulterant quantification.

Models	R_c_^2^	RMSEC (%)	R_p_^2^	RMSEP (%)
EML	0.9820	6.0730	0.9280	9.9885
LR	0.9451	10.4186	0.9541	8.2852
GPR	0.9892	4.6878	0.9570	7.6243

## Data Availability

Data is contained within the article.
